# Detection of Glutamic Acid in Oilseed Rape Leaves Using Near Infrared Spectroscopy and the Least Squares-Support Vector Machine

**DOI:** 10.3390/ijms131114106

**Published:** 2012-10-31

**Authors:** Yidan Bao, Wenwen Kong, Fei Liu, Zhengjun Qiu, Yong He

**Affiliations:** College of Biosystems Engineering and Food Science, Zhejiang University, Hangzhou 310058, China; E-Mails: ydbao@zju.edu.cn (Y.B.); zjukww@163.com (W.K.); zjqiu@zju.edu.cn (Z.Q.)

**Keywords:** oilseed rape, herbicide, amino acid, near infrared spectroscopy, successive projections algorithm, least squares-support vector machine

## Abstract

Amino acids are quite important indices to indicate the growth status of oilseed rape under herbicide stress. Near infrared (NIR) spectroscopy combined with chemometrics was applied for fast determination of glutamic acid in oilseed rape leaves. The optimal spectral preprocessing method was obtained after comparing Savitzky-Golay smoothing, standard normal variate, multiplicative scatter correction, first and second derivatives, detrending and direct orthogonal signal correction. Linear and nonlinear calibration methods were developed, including partial least squares (PLS) and least squares-support vector machine (LS-SVM). The most effective wavelengths (EWs) were determined by the successive projections algorithm (SPA), and these wavelengths were used as the inputs of PLS and LS-SVM model. The best prediction results were achieved by SPA-LS-SVM (Raw) model with correlation coefficient *r* = 0.9943 and root mean squares error of prediction (RMSEP) = 0.0569 for prediction set. These results indicated that NIR spectroscopy combined with SPA-LS-SVM was feasible for the fast and effective detection of glutamic acid in oilseed rape leaves. The selected EWs could be used to develop spectral sensors, and the important and basic amino acid data were helpful to study the function mechanism of herbicide.

## 1. Introduction

Oilseed rape (*Brassica napus* L.) is one of the most important sources of edible oil in India, North America, Europe, and China. Rapeseeds are also used as feeds and renewable energy for sustainable development of the world. In China, oilseed rape is expanding rapidly as a rotation crop following rice [1]. In order to keep a better and higher quality and quantity of rapeseeds, the growth environment should be suitable during oilseed rape growth stage. Along with the reducing number of labor available for farming, more chemical constituents are applied to remove the weeds, insects, and so on. Recently, a newly developed propyl 4-(2-(4,6-dimethoxypyrimidin-2-yloxy)benzylamino)benzoate (ZJ0273) has been applied to remove the weeds as an ALS (acetolactate synthase)-inhibiting herbicide, which is thought to be environmentally friendly. Amino acid is one of the most important parameters and basic data to indicate herbicide function mechanism during oilseed rape growth stages. Hence, the detection of amino acid is quite important. Amino acid is traditionally determined by amino acid analyzer or high performance liquid chromatography (HPLC). These methods were time consuming, laborious, costly and not suitable for fast determination and in field monitoring during the various growth stages of oilseed rape.

Nowadays, near infrared (NIR) spectroscopy is a widely applied promising technology for fast and environmental friendly determination method of plant growth information [2]. This technology has been applied in oilseed rape for information detection, such as the detection of nutritional information of rape leaves [3], acetolactate synthase (ALS), total amino acids, and protein content of oilseed rape leaves using visible/near infrared (400–1100 nm) spectra [4–6]. Glutamic acid is one of the major amino acids, and it is also an important composition of protein. Amino acids and protein can directly or indirectly influence plant metabolism, such as photosynthesis and respiration, which are important for plant growth. Furthermore, glutamic acid is one of the major suppliers of amino for the function of ketone of amino acid [7]. Hence, the glutamic acid concentration is quite important for the synthesis of amino acid. The determination of glutamic acid can supply useful basic data to study the influence of herbicide stress on oilseed rape growth, which is useful for field precise management and operations of oilseed rape. However, to our knowledge, there has been no report about the determination of glutamic acid under the herbicide ZJ0273 stress in oilseed rape using near-infrared spectroscopy within 1100–2500 nm.

The objective of this study was to develop a suitable method for fast determination of glutamic acids in oilseed rape leaves under herbicide stress using near infrared spectroscopy. Moreover, this study would determine the most effective wavelengths, and settle the optimal spectral preprocessing method and calibration method.

## 2. Results and Discussion

### 2.1. Spectral Features of Oilseed Rape

**Figure 1:**
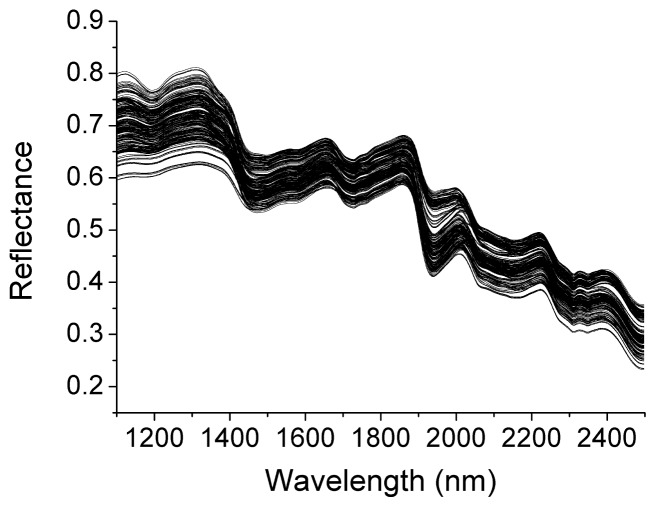
Raw reflectance spectra of oilseed rape leaves.

The raw reflectance spectra of oilseed rape leaves are shown in Figure 1. As can be seen, there were many peaks which might be corresponding to chemical compositions with C–H, N–H or O–H bands. These wavelength bands might be related to the physiological constituents in oilseed rape leaves under herbicide stress. These peaks and valleys might be sensitive and helpful to develop a correlationship between spectral data and amino acid in oilseed rape leaves.

The statistics of amino acid in oilseed rape leaves are shown in Table 1. As can be seen, the range of calibration set covered a larger range than validation set, which was thought to be helpful for the development of a robust calibration model. The reason for a relatively small difference in average and standard variation values was that the samples in these three sets all included the samples of three different collecting times, four herbicide treatment concentrations and five leaf positions. The developed model considered the major influences (treatment times, herbicide concentrations and leaf positions) of sample variance in this experiment.

### 2.2. The Performance of PLS Models

Before the PLS model, the reflectance spectra of leaf samples were treated by the aforementioned preprocessing methods, including SG, SNV, MSC, 1-der, 2-der, detrending and DOSC. Different PLS models were developed with preprocessed spectra and different latent variables (LVs). The calibration and validation sets were applied to develop a stable and robust model. The samples in prediction set were used to assess the prediction performance of developed models. According to the aforementioned evaluation standards, such as *r* and RMSEP, the optimal preprocessing method could be determined. The prediction results by PLS models are shown in Table 2. As shown in Table 2, the optimal performance was achieved by 1-der spectra with *r* = 0.9678 and RMSEP = 0.1335. Then followed good prediction performance was achieved by Raw and SG preprocessed spectra. In PLS models, 700 variables were employed as inputs which might contain much collinearity and redundancy between these wavelengths. In order to develop a more parsimonious model with fewer variables, an effective wavelength selection procedure should be performed for further analysis. Herein, SPA was investigated to extract the effective wavelengths.

### 2.3. EWs Selected by SPA

According to the prediction results of PLS models, the optimal preprocessing method was 1-der. Then the 1-der spectra were applied for effective wavelength selection. The raw spectra were also processed to extract effective wavelengths compared with 1-der spectra. During SPA procedure, the maximum number of selected variable was set as 30. The selected effective wavelengths should have least collinearity and redundancies, which would be helpful to develop a stable and robust models. The selected EWs by SPA are shown in Table 3. According to the principles of SPA, the EWs were extracted and sequenced in the order of importance for the prediction of amino acid. Taking raw spectra for instance, 2252 nm was the first selected wavelength, which meant 2252 nm was the most important wavelength for prediction of glutamic acid, then followed by 2228, 1404 nm, and so on. Wavelengths around 2252, 2266, and 2268 nm might be due to the N–H stretch and N–H in plane bending motion [8]. Wavelengths around 2426 nm might be attributed to the stretching and bending vibrations of the CH_2_ groups of the side chains of amino acids [9]. These EWs in raw and 1-der spectra were applied as inputs of LS-SVM to develop SPA-LS-SVM models, which was a newly proposed combination innovation as a high performance calibration method in previous study [10]. Then SPA-LS-SVM models were applied for the determination of glutamic acid in oilseed rape leaves.

### 2.4 PLS and LS-SVM Models Based on SPA

**Figure 2:**
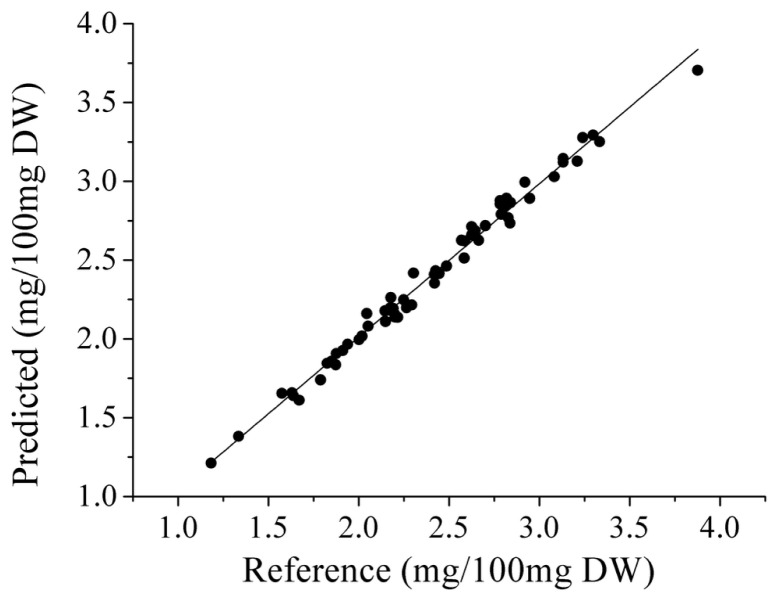
Reference *vs.* predicted values of glutamic acid by SPA-LS-SVM (Raw).

According to the aforementioned procedure, the SPA-LS-SVM models were developed and compared with SPA-PLS models. In SPA-PLS models, the selected EWs were applied to develop PLS models, and different latent variables (LVs) were used in these PLS models. In LS-SVM models, the EWs were used as the input variables. RBF kernel was used as kernel function, and the optimal combination of (γ, σ^2^) was determined by a two-step grid search technique. The search region of (γ, σ^2^) was set as 10^−2^–10^5^, which was determined according to experience and previous literature [4,11,12]. The optimal (γ, σ^2^) were (1.5 × 10^3^, 59.9) and (38.6, 29.5) for raw and 1-der spectra in SPA-LS-SVM models, respectively. The prediction results for prediction set by SPA-PLS and SPA-LS-SVM models are shown in Table 2. As can be seen, SPA-LS-SVM (Raw) model was the best one, which slightly outperformed SPA-PLS (Raw and 1-der) and SPA-LS-SVM (1-der) models. The prediction results by SPA-LS-SVM (Raw) were *r* = 0.9943 and RMSEP = 0.0569. The RPD value for calibration model was also the highest one (12.2), which indicated that SPA-LS-SVM (Raw) model had the best prediction performance. The scatter plots of prediction set by SPA-LS-SVM (Raw) are shown in Figure 2. Comparing all developed models (seen in Table 2), the best model was SPA-LS-SVM (Raw) model with an excellent prediction precision. The reason for better performance might be that LS-SVM could handle both linear and nonlinear correlation problems. The results also indicated that SPA was a powerful method to select significant and effective wavelengths. SPA also reduced the computing complexity and cost less time, and improved the effectiveness and precision of developed models. The overall results indicated that NIR spectroscopy combined with SPA-LS-SVM could be applied for the fast and accurate determination of glutamic acid in oilseed rape leaves under herbicide stress. This study proposed a new direction to use NIR for in field monitoring of growth status and physiological parameters of oilseed rape.

## 3. Materials and Methods

### 3.1. Sample Preparation

The oilseed rape (*Brassica napus*, cv. ZS758) was planted at the farm of Zhejiang University, Hangzhou (30°10′N, 120°12′E). Four different concentrations of herbicide ZJ0273 (0, 100, 200 and 500 mg/L) were foliar applied at 5-leaf stage at the quantity of 500 L/ha. The 0 mg/L concentration was used as contrast test, which means no herbicide was used in this concentration level. Conventional crop management was used during the growth period. The experiment was implemented in the seedling stage of oilseed rape. The plants in the central rows of each plot were used for the physiological measurement at three separate times (7, 14 and 28 d after ZJ0273 treatment). The whole plants were directly collected in field, and the fresh leaves in different leaf positions were separated and labeled as different samples. The leaf position played a more important role to influence the content of amino acids under herbicide stress (ZJ0273). Even in one plant, the leaves with different leaf positions had an obvious difference of amino acid content. 80, 80 and 88 samples were collected for 7, 14 and 28 d after herbicide treatment, respectively. Hence, a total of 248 samples were collected after the herbicide treatment. The collected samples were firstly dried in an oven at 60 °C for 2 d, and then ground in an electric mill using Universal High-Speed Smashing Machines (Model: FW100, Tianjin City Taisite Instrument Co., LTD, Tianjin, China). The milled samples were sieved through 60-mesh in order to reduce the influence of different particle sizes. Then the samples were randomly separated into calibration (124 samples), validation (62 samples) and prediction (62 samples) sets. The calibration and validation sets were utilized to develop a stable and robust model, and the validation set played the role of test set as cross-validation method. The prediction set was used for prediction performance assessment.

### 3.2. Spectral Collection and Reference Method

Foss NIRSystems 5000 (Foss NIRSystems, Hillerød, Denmark) was applied for near infrared reflectance spectral collection within 1100–2500 nm. The samples were firstly taken out from the refrigerator until the sample temperature reached room temperature at 20–23 °C. The room humidity was around 50%–60%. The cell used in the experiment was with a diameter of 1 cm. The standard sample supplied by Foss Company was used to calibrate the Foss NIRSystems 5000 spectrometer. The resolution of instrument was 2 nm, and 700 data points were collected for each sample. Three replicative spectra were collected for each sample and the averaged spectrum was used as the spectra of each sample.

The glutamic acid was determined by HITACHI amino acid analyzer (Model: L-8900, Tokyo, Japan). This experiment and calibration was specific for glutamic acid, not a fairly constant proportion of total protein content. The chemical pretreatment and operation steps of oilseed rape leaf were based on the Lisiewska method [13]. The analyzer was implemented under a normal analytical condition. Two glutamic acid values were obtained for each sample, and the averaged value was used as the reference value of each sample. The content of amino acids was expressed as mg/100 mg of dry matter (mg/100 mg DW).

### 3.3. Spectral Preprocessing and SPA

Normally, there were some noise and undesired disturbance of instrument or environment in the spectral data obtained for oilseed rape leaf samples. For a stable and acceptable prediction model, some spectral preprocessing methods were investigated before model development. These methods were applied to remove the spectral baseline shift, noise and light scatter influence [14]. The processing of spectra were proceeded by “The Unscrambler V9.8” (CAMO AS, Oslo, Norway), including Savitzky-Golay smoothing (SG), standard normal variate (SNV), multiplicative scatter correction (MSC), first-derivative (1-der), second-derivative (2-der) and detrending. Another method, named direct orthogonal signal correction (DOSC), was implemented by Matlab (version 7.0; The Math Works: Natick, MA, USA, 2004).

In order to reduce the computing time and achieve a parsimonious model, some informative wavelengths were selected as effective wavelengths (EWs). These EWs were used as inputs of calibration methods instead of full wavelength region within 1100–2500 nm. Herein, successive projections algorithm (SPA) was used for the most effective wavelength selection. SPA could select the most effective wavelength with least collinearity and redundancies though the projection procedure, and the details of SPA algorithm could be found in the literature [15,16].

### 3.4. PLS and LS-SVM Methods

Partial least squares (PLS) analysis is a widely utilized calibration method for regression modeling. The main procedure is to extract the PLS factors and develop a linear correlationship between the PLS factors and chemical constituents (glutamic acid). The details of PLS could be found in the references [17].

Least squares-support vector machine (LS-SVM) is a promising method for both linear and nonlinear regression analysis, which costs less time and requires a small sample database. The details of LS-SVM could be found in the literature [11,18]. LS-SVM was applied to develop the correlation between the selected EWs by SPA and amino acid in oilseed rape leaves under herbicide stress. Before the development of LS-SVM model, the input variables, kernel function and model parameters should be firstly determined. In this study, the inputs were the selected EWs by SPA, the kernel function was the radial basis function (RBF), and the model parameters gamma (γ) and sig2 (σ^2^) were determined by a two-step grid search technique. All the calculations were performed using Matlab V7.0. The free LS-SVM V1.5 toolbox (Suykens, Leuven, Belgium) was applied with Matlab V7.0 to develop the LS-SVM models.

The main evaluation indices for PLS and LS-SVM models were correlation coefficients (*r*), root mean squares error of prediction (RMSEP) and residual predictive deviation (RPD). RPD was used to evaluate how well the calibration model could predict compositional data [12]. The optimal models should have a higher correlation coefficients (closer to 1) and lower RMSEP values.

## 4. Conclusions

Near infrared spectroscopy combined with SPA-LS-SVM was successfully applied for the fast determination of glutamic acid in oilseed rape leaves under herbicide ZJ0273 stress. This study supplied a fast method for the acquisition of basic physiological parameter data to study the function mechanism and metabolic way of herbicide. The optimal preprocessing method for this specific study was 1-der by PLS models. The optimal prediction results were obtained by SPA-LS-SVM (Raw) model using EWs with *r* = 0.9943 and RMSEP = 0.0569. The result indicated that NIR combined with SPA-LS-SVM could be applied for glutamic acid detection. After knowing the glutamic acid in a rapid and convenient way using NIR spectroscopy, it could be used to monitor the growth status and herbicide effects of oilseed rape. The selected effective wavelengths by SPA could be used for the development of portable spectral instruments or in field monitoring sensors equipped on field operation machines to detect plant growth status. Based on this study method and further research of lab and field experiments, we could understand the degree of influence that herbicide stress has on oilseed rape. We could apply a proper amount of herbicide to prevent weeds effectively, while not influencing the normal growth of oilseed rape. This study supplied a helpful direction for weed control and management, growth status monitoring and operations.

## Figures and Tables

**Table 1:** Statistics of glutamic acid content of oilseed rape leaves.

Set	No.	Range (mg/100 g DW)	Mean (mg/100 g DW)	S.D. (mg/100 g DW)
Calibration	124	1.176–4.101	2.410	0.5252
Validation	62	1.189–3.654	2.411	0.5219
Prediction	62	1.183–3.877	2.411	0.5313

**Table 2:** Prediction results of glutamic acid by partial least squares (PLS) and successive projections algorithm-least squares-support vector machine (SPA-LS-SVM) models.

Model	Treatment	LV/EW/(γ, σ^2^)	Calibration		Validation		Prediction		RPD

			*R_c_*	RMSEC	*R_v_*	RMSEV	*R_p_*	RMSEP	
PLS	Raw	8/700/-	0.9474	0.1674	0.9603	0.1462	0.9591	0.1530	3.6
	SG	8/700/-	0.9471	0.1678	0.9602	0.1463	0.9591	0.1528	3.6
	SNV	7/700/-	0.9414	0.1765	0.9542	0.1552	0.9519	0.1628	3.4
	MSC	7/700/-	0.9413	0.1765	0.9546	0.1546	0.9524	0.1621	3.4
	1-Der	6/700/-	0.9629	0.1412	0.9694	0.1282	0.9678	0.1335	4.1
	2-Der	4/700/-	0.9603	0.1459	0.9585	0.1483	0.9576	0.1527	3.5
	Detrending	7/700/-	0.9507	0.1623	0.9598	0.1463	0.9550	0.1571	3.6
	DOSC	4/700/-	0.9361	0.1840	0.9460	0.1692	0.9436	0.1752	3.1
SPA-PLS	Raw	8/19/-	0.9490	0.1649	0.9607	0.1458	0.9557	0.1591	3.6
	1-Der	3/10/-	0.9487	0.1654	0.9575	0.1501	0.9528	0.1608	3.5
SPA-LS-SVM	Raw	-/19/ (1.5 × 10^4^, 59.9)	0.9911	0.0700	0.9966	0.0431	0.9943	0.0569	12.2
	1-Der	-/10/ (38.6, 29.5)	0.9869	0.0846	0.9952	0.0514	0.9787	0.1100	10.2

**Table 3:** Selected effective wavelengths (EWs) by SPA.

Preprocessing	No.	Selected EWs (nm)
Raw	19	2252, 2228, 1404, 2268, 2178, 1434, 2426, 1844, 1692, 1190, 1344, 1636, 1730, 1892, 1234, 1546, 2409, 2046, 1776
1-Der	10	1678, 2266, 2486, 2234, 2296, 1272, 1534, 2444, 2208, 1718
